# Personalized absolute benefit of statin treatment for primary or secondary prevention of vascular disease in individual elderly patients

**DOI:** 10.1007/s00392-016-1023-8

**Published:** 2016-08-23

**Authors:** Manon C. Stam-Slob, Frank L. J. Visseren, J. Wouter Jukema, Yolanda van der Graaf, Neil R. Poulter, Ajay Gupta, Naveed Sattar, Peter W. Macfarlane, Patricia M. Kearney, Anton J. M. de Craen, Stella Trompet

**Affiliations:** 1 Department of Vascular Medicine, University Medical Center Utrecht, Heidelberglaan 100, 3584 CX Utrecht, The Netherlands; 2 Department of Cardiology, Leiden University Medical Center, Albinusdreef 2, 2333 ZA Leiden, The Netherlands; 3 Julius Center for Health Sciences and Primary Care, University Medical Center Utrecht, Universiteitsweg 100, 3584 CG Utrecht, The Netherlands; 4 Department of Vascular Medicine, ICCH, Imperial College London, White City, London, W12 0NN UK; 5 Institute of Cardiovascular and Medical Sciences, Cardiovascular Research Centre, University of Glasgow, Glasgow, G12 8TA UK; 6 Department of Epidemiology and Public Health, University College Cork, Western Rd, Cork, Ireland; 7 Department of Gerontology and Geriatrics, Leiden University Medical Center, Albinusdreef 2, 2333 ZA Leiden, The Netherlands; 8 University Medical Center Utrecht, F02.224, P.O. Box 85500, 3508 GA Utrecht, The Netherlands

**Keywords:** Elderly, Statin, Absolute treatment effect, Vascular disease

## Abstract

**Objective:**

To estimate the absolute treatment effect of statin therapy on major adverse cardiovascular events (MACE; myocardial infarction, stroke and vascular death) for the individual patient aged ≥70 years.

**Methods:**

Prediction models for MACE were derived in patients aged ≥70 years with (*n* = 2550) and without (*n* = 3253) vascular disease from the “PROspective Study of Pravastatin in Elderly at Risk” (PROSPER) trial and validated in the “Secondary Manifestations of ARTerial disease” (SMART) cohort study (*n* = 1442) and the “Anglo-Scandinavian Cardiac Outcomes Trial-Lipid Lowering Arm” (ASCOT-LLA) trial (*n* = 1893), respectively, using competing risk analysis. Prespecified predictors were various clinical characteristics including statin treatment. Individual absolute risk reductions (ARRs) for MACE in 5 and 10 years were estimated by subtracting on-treatment from off-treatment risk.

**Results:**

Individual ARRs were higher in elderly patients with vascular disease [5-year ARRs: median 5.1 %, interquartile range (IQR) 4.0–6.2 %, 10-year ARRs: median 7.8 %, IQR 6.8–8.6 %] than in patients without vascular disease (5-year ARRs: median 1.7 %, IQR 1.3–2.1 %, 10-year ARRs: 2.9 %, IQR 2.3–3.6 %). Ninety-eight percent of patients with vascular disease had a 5-year ARR ≥2.0 %, compared to 31 % of patients without vascular disease.

**Conclusions:**

With a multivariable prediction model the absolute treatment effect of a statin on MACE for individual elderly patients with and without vascular disease can be quantified. Because of high ARRs, treating all patients is more beneficial than prediction-based treatment for secondary prevention of MACE. For primary prevention of MACE, the prediction model can be used to identify those patients who benefit meaningfully from statin therapy.

**Electronic supplementary material:**

The online version of this article (doi:10.1007/s00392-016-1023-8) contains supplementary material, which is available to authorized users.

## Introduction

Vascular disease in the elderly (age ≥70 years) accounts for a high global burden of disease as risk of atherosclerotic vascular events and their case-fatality rate increase exponentially with age [1–3]. Older patients who survive a major adverse cardiovascular event (MACE) are commonly chronically disabled because of heart failure, cardiac arrhythmia or neurologic deficits. Population aging and the chronic complications of vascular events that physicians encounter in the elderly have triggered a debate about the benefit of cardiovascular risk management in these patients. The “PROspective Study of Pravastatin in Elderly at Risk” (PROSPER) trial found a 15 % reduction in myocardial infarction, stroke and coronary heart disease death with pravastatin treatment in elderly subjects [4]. Older patients are underrepresented in other trials evaluating cardiovascular prevention strategies and it is, therefore, uncertain whether statins are effective in the elderly. They have lower life-expectancy in general than middle-aged individuals which could potentially limit their benefit from statins. The benefit of statins is particularly uncertain and debated in those with limited life expectancy due to nonvascular diseases [5].

Statin therapy is recommended for the secondary prevention of MACE in those who have vascular disease, unless comorbidity and polypharmacy confound management [6–8]. For the primary prevention of MACE, the European guideline states that ‘statin therapy may be considered particularly in the presence of at least one other risk factor than age’ and the National Institute for Health and Care Excellence (NICE) guideline advices statin therapy in those with an estimated 10-year risk of MACE ≥10 % [7, 9]. Since the QRISK2 score estimates a 10-year risk ≥10 % for every patient aged ≥70 years and almost all older patients have at least one vascular risk factor besides age, statin therapy would be indicated for nearly all elderly patients [10]. However, under-prescription of statins in daily practice may in part reflect uncertainty about the extent to which elderly patients may benefit from a statin [11]. As the absolute risk for vascular disease and the absolute risk reduction (ARR) caused by a statin are influenced by individual patient characteristics, there is a potential range in the benefit received from a statin. Therefore, we aimed to estimate the absolute treatment effect of statin therapy on MACE for the individual elderly patient by developing and validating a prediction model based on individual patient characteristics [12, 13].

## Materials and methods

### Study populations

We developed two separate prediction models for MACE in patients with and without vascular disease aged ≥70 years, since a history of vascular disease is the strongest predictor for MACE in elderly subjects with great differences in risk profiles of elderly subjects with and without vascular disease. Moreover, guidelines differentiate between primary and secondary prevention of MACE [6, 7]. Both models were derived in the PROSPER trial population. The model for patients with vascular disease was validated in the “Secondary Manifestations of ARTerial disease” (SMART) cohort study and the model for patients without vascular disease in the “Anglo-Scandinavian Cardiac Outcomes Trial- Lipid Lowering Arm” (ASCOT-LLA) trial. The design and patient populations of these studies have been described in detail in the original publications [4, 14, 15]. Ethical approval was obtained for these studies. The PROSPER study included patients 70–82 years of age from Scotland, Ireland and the Netherlands with vascular disease or a high risk profile for vascular disease between 1997 and 1999. Patients were randomly assigned to 40 mg pravastatin per day or placebo. Patients from the elderly ASCOT-LLA population recruited between 1998 and 2000 were 70–79 years of age and were known to have hypertension (untreated or treated), but total cholesterol levels ≤6.5 mmol/l, in combination with three additional risk factors for vascular disease. They originated from the United Kingdom, Ireland and the Nordic Countries. Study participants were randomly assigned to atorvastatin 10 mg or placebo. All patients from the PROSPER and ASCOT-LLA trial were not taking a statin at the time of study inclusion. Elderly patients from the single-center prospective, observational SMART cohort study with a history of vascular disease from the Netherlands were 70–82 years of age and followed up between 1996 and 2014.

### Model derivation

We derived prediction models in the PROSPER trial for the combined outcome of myocardial infarction, stroke and vascular death (MACE) in elderly patients with (*n* = 2550) and without (*n* = 3253) vascular disease. Vascular disease included current or prior coronary artery disease (myocardial infarction, angina, coronary artery bypass graft/percutaneous coronary intervention), cerebrovascular disease (stroke or transient ischemic attack) or peripheral artery disease (claudication or peripheral artery surgery). We built a Fine & Gray competing risks model to account for nonvascular deaths [16]. Prespecified predictors from existing risk scores in the elderly were: sex, age, current smoking, diabetes, systolic blood pressure, low density lipoprotein (LDL)-cholesterol, high-density lipoprotein (HDL)-cholesterol, glomerular filtration rate (eGFR) and number of medications taken [17–19]. Variable selection was not applied to prevent optimism, which is the phenomenon that a model optimally fits the data in which it is derived, but is not generalizable to an external population. Glomerular filtration rate was assessed with the Modification of Diet in Renal Disease (MDRD) formula [20]. Polyvascular disease (vascular disease at ≥1 of the defined locations) was added as a predictor to the model for recurrent MACE. The number of medications per patient was included as a measure of comorbidity, not taking into account nasal sprays and topical skin medicines. Allocated statin treatment was added to both models. Statin treatment effect for secondary prevention of MACE was derived from the PROSPER population. For primary prevention of MACE, statin treatment effect was estimated in a pooled analysis of the PROSPER and ASCOT trial population, adjusted for potential study differences regarding statin type, patient population and clinical setting. This was done in a competing risks analysis of the pooled PROSPER and ASCOT-LLA individual patient data, with statin treatment and the trial patients originated from as independent variables. We singly imputed missing values by weighted probability matching using multivariate regression, as complete case analysis leads to loss of information and possibly to bias of coefficients [21]. Missing values were imputed for eGFR (*n* = 8, 0.1 %). Continuous predictors were truncated at the 1st and 99th percentile to minimize the influence of outliers in the model [22]. Whether the association of continuous predictors with the outcome variable was linear or not was assessed with restricted cubic splines [23].

Model performance was assessed with the c-statistic [95 % confidence interval (CI)] for discrimination and with calibration plots of predicted versus observed risk. The model was fitted for the prediction of 3.2-year risk (median follow-up). These estimations were extrapolated to derive 5-year and 10-year vascular event risks. An individual 5-year and 10-year ARR was estimated for each patient, by subtracting the predicted risk for a specific patient with statin treatment from his or her predicted risk without statin treatment (ARR = individual MACE risk without a statin−individual MACE risk with a statin). One can estimate the MACE risk and ARR with and without statin treatment for each individual patient by filling in patient characteristics in the model formula (Table S1). This ARR can be translated into an individual number needed to treat (iNNT), the number of patients with the exact same risk profile needed to treat to prevent 1 event in 5 or 10 years, respectively (iNNT = 100/ARR). For example, an estimated 5-year absolute risk reduction of 2 % means that one has to treat 50 patients with the exact same risk profile for 5 years to prevent 1 event [iNNT = 100/2 (ARR) = 50]. The distribution of MACE risk and ARR in patients with and without vascular disease is presented in a histogram and described as median with an interquartile range (IQR).

### Model validation

The derived model for patients with vascular disease was externally validated in the SMART cohort study (*n* = 1442) and the model for patients without vascular disease in the ASCOT-LLA trial (*n* = 1893). Discrimination was assessed with the c-statistic (95 % CI) and calibration with plots of predicted versus observed risk. To optimally estimate vascular risk and treatment effect for individual patients we adjusted for geographic differences by recalibrating the models with updated cumulative baseline hazard and mean linear predictor, while effect sizes of predictors did not change. Missing values in the ASCOT-LLA trial were imputed for creatinine (*n* = 54, 2.9 %), LDL-cholesterol (*n* = 185, 9.8 %) and number of medications (*n* = 1156, 61 %). In the SMART study, missing values were imputed for systolic blood pressure (*n* = 11, 0.8 %), LDL-cholesterol (*n* = 37, 2.7 %), HDL-cholesterol (*n* = 13, 0.9 %), eGFR (*n* = 4, 0.3 %) and smoking (*n* = 10, 0.7 %). We estimated baseline LDL-cholesterol concentrations for patients in the SMART cohort study who were already on a statin at the time of study inclusion, according to the expected LDL-cholesterol reduction that the different statin preparations with their dosages probably had achieved [24].

### Sensitivity analyses

We performed a sensitivity analysis to assess what the expected individual ARR would be if patients were treated with atorvastatin 20 mg as recommended by the NICE guideline for primary prevention of vascular disease [9]. We assumed that atorvastatin 20 mg gives 6 % more LDL-cholesterol reduction than pravastatin 40 mg or atorvastatin 10 mg. In this scenario, the relative risk reduction with a statin would be 25 % instead of 22 % for secondary prevention and 15 % instead of 13 % for primary prevention of vascular disease. In a second sensitivity analysis, individual ARRs for primary prevention of MACE were estimated with a combined statin relative risk reduction of 16 % for patients aged ≥75 years from different trial populations [25].

### Net benefit analysis

The different treatment strategies (treating none, treating all patients and treating patients according to the prediction model with a statin) were compared with each other in a net benefit analysis [26]. This method shows whether it is valid to base treatment decisions on the prediction model. Methods and results (Fig. S1, Table S2) can be found in the Supplementary material.

Analyses were performed in R statistical software 3.2.0 with the add-on packages rms, plyr, pec, riskRegression, and cmprsk (extended by Wolbers et al. [16]).

## Results

### Patient population and trial outcomes

The study population consisted of elderly patients with vascular disease (PROSPER *n* = 2550, SMART *n* = 1442) and patients without vascular disease (PROSPER *n* = 3253, ASCOT-LLA *n* = 1893). Baseline characteristics are presented in Table 1. Mean age of patients with vascular disease was 75.7 [standard deviation (SD) 3.4] years in the PROSPER trial and 73.6 (SD 2.7) years in the SMART study. Mean age of patients without vascular disease was 75.1 (SD 3.3) years in the PROSPER trial and 74.1 (SD 2.7) years in the ASCOT-LLA trial. During a median follow-up of 3.2 years in patients with vascular disease from the PROSPER trial, 517 MACE [68/1000 person years (PY)] and 114 nonvascular deaths occurred. Median follow-up in SMART patients with vascular disease was 5.4 years with 398 MACE (46/1000 PY) and 212 nonvascular deaths. Patients without vascular disease from the PROSPER trial experienced 395 MACE (39/1000 PY) and 155 nonvascular deaths. In ASCOT-LLA patients without vascular disease, median follow-up was 3.1 years with 128 MACE (22/1000 PY) and 86 nonvascular deaths.

**Table 1:** Baseline characteristics of elderly patients (age ≥70 years) with and without vascular disease

	With vascular disease		Without vascular disease	
	PROSPER (*n* = 2550)	SMART (*n* = 1442)	PROSPER (*n* = 3253)	ASCOT-LLA (*n* = 1893)
Demographics				
Male gender (*n*, %)	1453 (57.0)	1062 (73.7)	1350 (41.5)	1525 (80.6)
Age (years)	75.7 (3.4)	73.6 (2.7)	75.1 (3.3)	74.1 (2.7)
Country of residence (*n*, %)				
Scotland/UK^a^	1232 (48.3)		1288 (39.6)	990 (52.3)
Ireland	845 (33.1)		1338 (41.1)	19 (1.0)
The Netherlands	473 (18.5)	1442 (100)	627 (19.3)	
Denmark				133 (7.0)
Finland				126 (6.7)
Iceland				11 (0.6)
Norway				205 (10.8)
Sweden				409 (21.6)
Current smoker (*n*, %)	474 (18.6)	238 (16.5)	1084 (33.3)	402 (21.2)
*N* medications (median, IQR)	4 (3–6)	5 (4–7)	3 (2–4)	2 (1–3)
Statin treatment (*n*, %)	1299 (50.9)	885 (61.4)	1591 (48.9)	939 (49.6)
Medical history				
Diabetes (*n*, %)	224 (8.7)	293 (20.3)	399 (12.3)	514 (27.2)
Cardiovascular disease (*n*, %)				
Coronary artery disease	1524 (59.8)	610 (42.3)	0 (0)	0 (0)
Cerebrovascular disease	425 (16.7)	276 (19.1)	0 (0)	0 (0)
Peripheral artery disease	206 (8.1)	238 (16.5)	0 (0)	0 (0)
Polyvascular disease	395 (15.5)	318 (22.1)	0 (0)	0 (0)
Physical examination				
Heart rate (beats/min)	65.2 (11.6)	66.0 (14.1)	67.2 (11.6)	69.6 (12.3)
Systolic blood pressure (mmHg)	152 (22)	148 (22)	157 (22)	170 (19)
Laboratory measurements				
LDL cholesterol (mmol/l)	3.8 (0.8)	3.8 (1.2)	3.8 (0.8)	3.5 (0.7)
HDL cholesterol (mmol/l)	1.2 (0.3)	1.3 (0.4)	1.3 (0.4)	1.3 (0.4)
eGFR (ml/min/1.73 m^2^)	58.8 (14.3)	66.8 (16.7)	61.0 (14.7)	63.6 (11.8)

Data are displayed as mean (SD) unless indicated otherwise

^a^ *UK* United Kingdom

### Model derivation and performance for patients with vascular disease

**Fig. 1:**
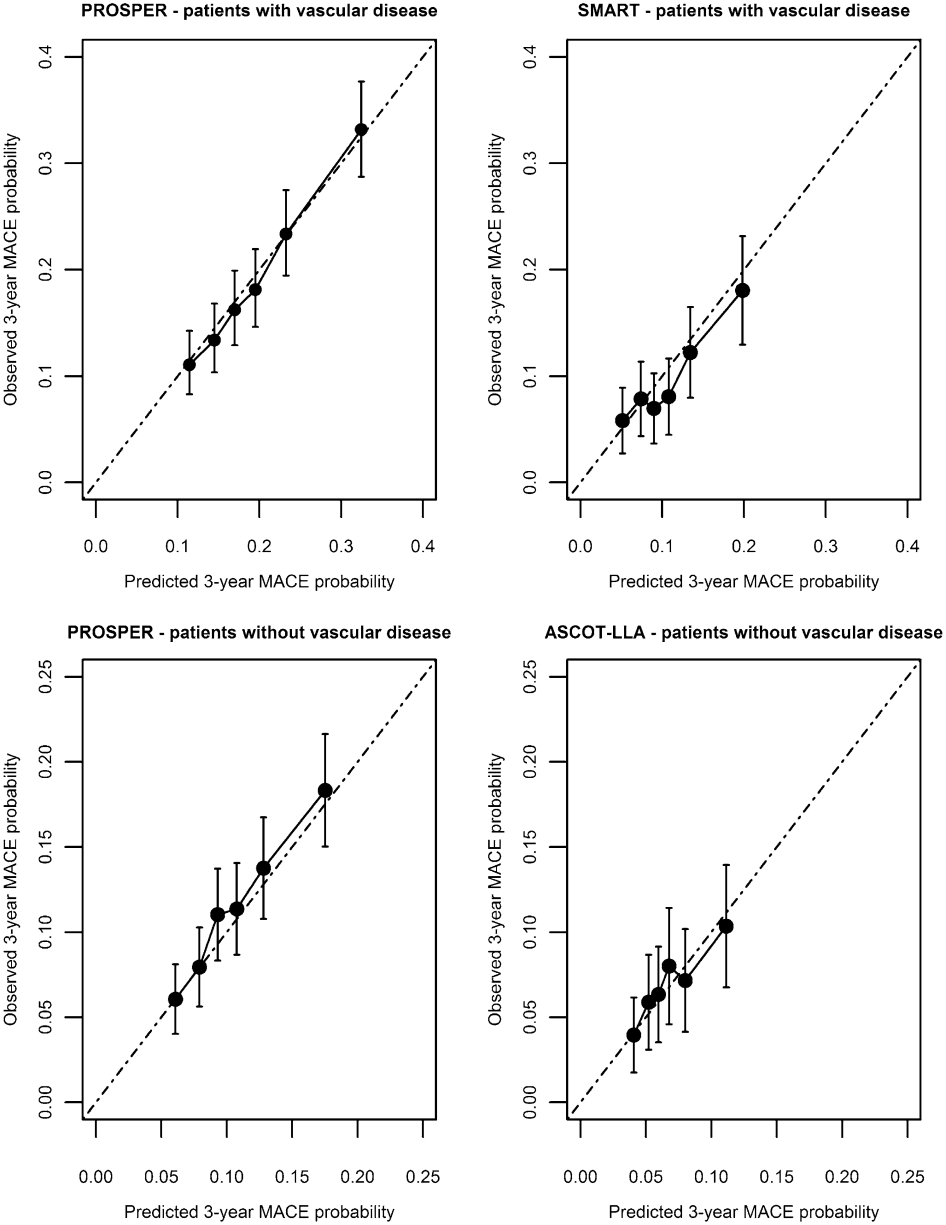
Calibration plots of predicted versus observed MACE risk in elderly patients

The derived model in patients with vascular disease is presented in Table 2A. Baseline systolic blood pressure and LDL-cholesterol were exponentially related to the outcome. LDL-cholesterol was not a major independent predictor for MACE. There was no interaction present between statin treatment and baseline risk, baseline LDL-cholesterol, LDL-cholesterol after 3 months of randomisation, age, renal function or polyvascular versus monovascular disease (*p* values >0.2). Model performance in the derivation set showed a good calibration (Fig. 1) and moderate discrimination [c-statistic 0.62 (95 % CI 0.60–0.64)]. After recalibration, the model calibrated well in the SMART validation set (Fig. 1) with a moderate discriminative performance [c-statistic 0.60 (95 % CI 0.56–0.63)].

**Table 2:** Fitted prediction models for major adverse cardiovascular events in elderly patients

Variable	Coefficient	sHR (95 % CI)	*p* value
A. Patients with vascular disease			
Male sex	0.401	1.49 (1.21–1.84)	<0.001
Age (years)	0.042	1.04 (1.02–1.07)	0.002
Current smoking	0.240	1.27 (1.02–1.58)	0.031
Diabetes	0.543	1.72 (1.31–2.26)	<0.001
Polyvascular disease	0.344	1.41 (1.13–1.76)	0.003
Number of medications	0.053	1.06 (1.01–1.10)	0.009
Systolic blood pressure (per 10 mmHg)	−0.366		0.084
Systolic blood pressure (per 10 mmHg)^2^	0.001		0.084
LDL-cholesterol (mmol/l)	0.876		0.074
LDL-cholesterol (mmol/l)^2^	−0.109		0.080
HDL-cholesterol (mmol/l)	0.081	1.08 (0.82–1.43)	0.570
eGFR (per 10 ml/min/1.73 m^2^)	−0.053	0.95 (0.88–1.02)	0.150
Statin treatment	−0.245	0.78 (0.66–0.93)	0.006
B. Patients without vascular disease			
Male sex	0.283	1.33 (1.06–1.66)	0.013
Age (years)	0.037	1.04 (1.01–1.07)	0.018
Current smoking	0.290	1.34 (1.07–1.68)	0.012
Diabetes	0.210	1.23 (0.93–1.64)	0.150
Number of medications	0.090	1.09 (1.05–1.15)	<0.001
Systolic blood pressure (per 10 mmHg)	0.060	1.06 (1.01–1.11)	0.014
LDL-cholesterol (mmol/l)	0.007	1.01 (0.89–1.15)	0.920
HDL-cholesterol (mmol/l)	−0.359	0.70 (0.51–0.96)	0.028
eGFR (per 10 ml/min/1.73 m^2^)	−0.613		0.008
eGFR (per 10 ml/min/1.73 m^2^)^2^	0.005		0.008
Statin treatment	−0.140	0.87 (0.73–1.03)	0.110

Models derived with Fine and Gray competing risk analysis

*sHR* subdistribution hazard ratio, *CI* confidence interval

### Model derivation and performance for patients without vascular disease

The derived model for patients without vascular disease is presented in Table 2B. Renal function (eGFR) was exponentially related to the outcome. LDL-cholesterol was no independent risk factor for MACE. There was no interaction present between statin treatment and baseline risk, baseline LDL-cholesterol, LDL-cholesterol after 3 months of randomisation or age (*p* values >0.4). An interaction between statin treatment and eGFR (*p* = 0.006) in the derivation set was not present in the validation set and, therefore, not included in the model. The model calibrated well in the derivation set (Fig. 1) with a moderate discriminative performance [c-statistic 0.61 (95 % CI 0.58–0.63)]. After recalibration, model calibration was good in the ASCOT-LLA validation set (Fig. 1) with a low discriminative performance [c-statistic 0.57 (95 % CI 0.53–0.63)].

### Five-year and ten-year predicted absolute risk for MACE and the absolute risk reduction if treated with a statin

**Fig. 2:**
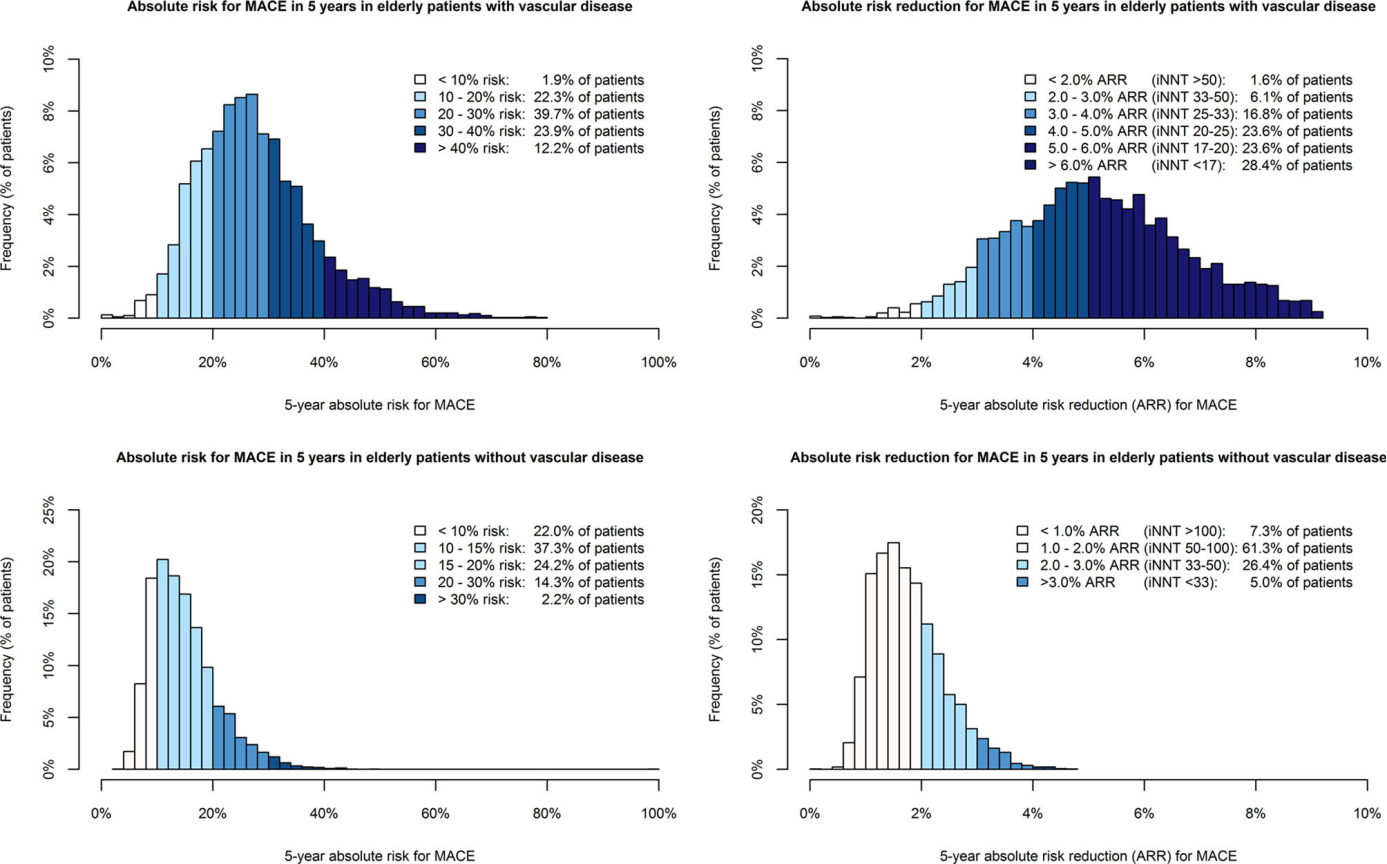
Distribution of 5-year absolute risk for MACE and the absolute risk reduction with statin therapy in elderly patients

**Fig. 3:**
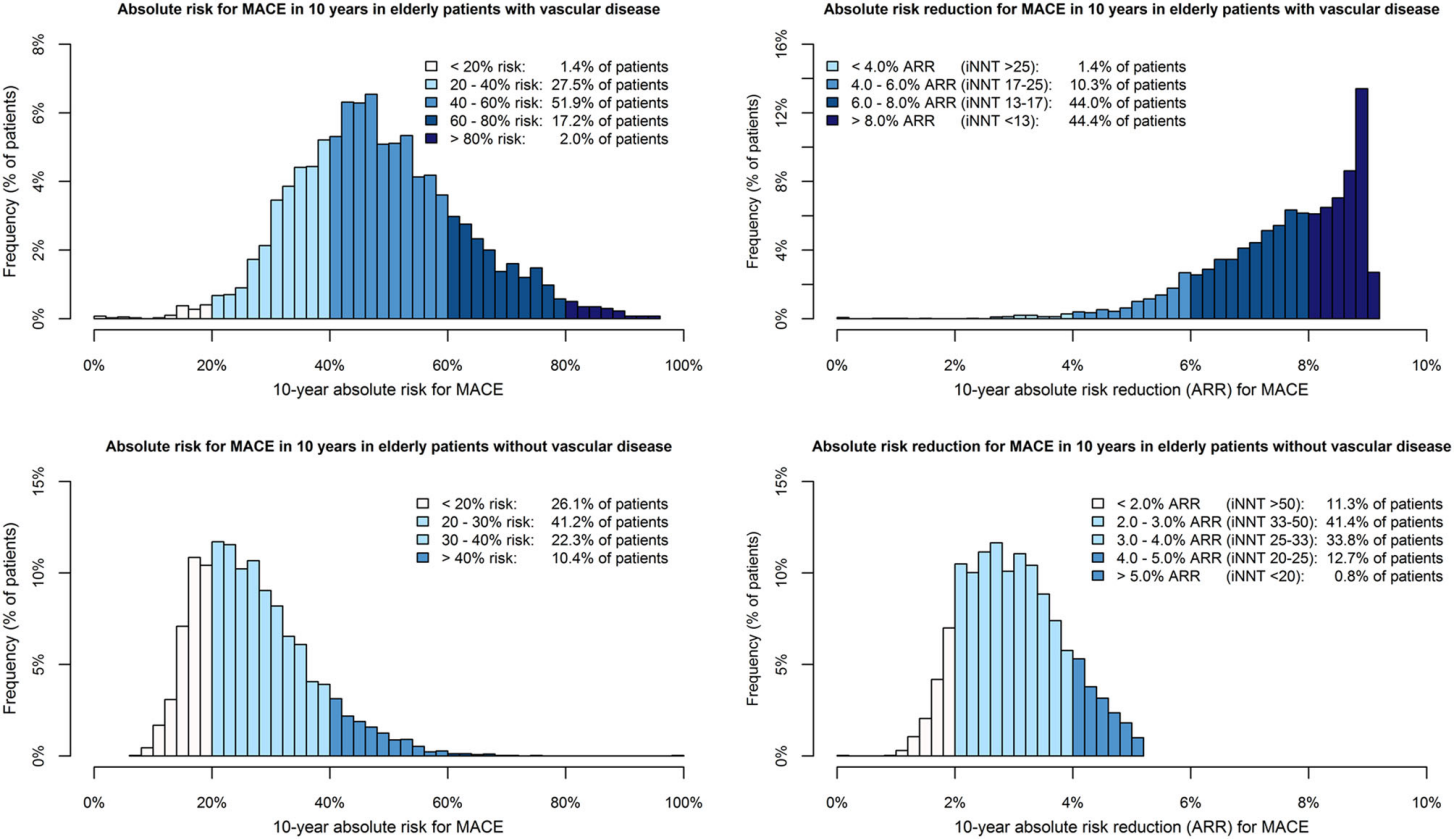
Distribution of 10-year absolute risk for MACE and the absolute risk reduction with statin therapy in elderly patients

Figures 2 and 3 show the distribution of 5-year and 10-year MACE risk and the absolute risk reductions in patients with vascular disease from the PROSPER trial and SMART study, and in patients without vascular disease from the PROSPER and ASCOT-LLA trials. There was a wide distribution of MACE risk in patients with vascular disease (5-year: median 26.4 %, IQR 20.3–33.6 %, 10-year: median 46.9 %, IQR 38.5–57.0 %) and in those without vascular disease (5-year: median 13.7 %, IQR 10.4–17.8 %, 10-year: median 25.5 %, IQR 19.8–32.4 %). Individual 5-year ARRs with a statin were higher in patients with vascular disease (median 5.1 %, IQR 4.0–6.2 %) than in patients without vascular disease (median 1.7 %, IQR 1.3–2.1 %). Ninety-eight percent of patients with vascular disease had a 5-year ARR ≥2.0 % (iNNT ≤50), compared to 31 % of patients without vascular disease. In patients with vascular disease the median 10-year ARR was 7.8 % (IQR 6.8–8.6 %) compared to a median 10-year ARR of 2.9 % (IQR 2.3–3.6 %) in patients without vascular disease.

### Sensitivity analyses

Under the assumption that atorvastatin 20 mg lowers LDL-cholesterol with an additional 6 % compared to pravastatin 40 mg or atorvastatin 10 mg, the median 5-year ARR would be 6.0 % (IQR 4.7–7.3 %) in patients with vascular disease and 2.0 % (IQR 1.5–2.5 %) in patients without vascular disease. Forty-nine percent of patients without vascular disease would have a 5-year ARR ≥2.0 %. Median 10-year ARR would be 9.3 % (IQR 8.1–10.2 %) for patients with vascular disease and 3.5 % (IQR 2.8–4.2 %) for patients without vascular disease.

Assuming statin therapy reduces MACE by 16 % in patients without vascular disease, the median 5-year ARR would be 2.1 % (IQR 1.6–2.6 %) and the median 10-year ARR would be 3.6 % (IQR 2.9–4.4 %). Fifty-three percent of patients without vascular disease would have a 5-year ARR ≥2.0 %.

## Discussion

Risk for MACE and the absolute treatment effect of a statin on MACE for individual elderly patients can be estimated with a clinical prediction model containing simple, readily available patient characteristics. There is a wide distribution of MACE risk in elderly patients with and without vascular disease. For secondary prevention of MACE, treating all patients is most beneficial since predicted absolute risk reductions are almost invariably high. With the use of a prediction model that quantifies an individual’s expected absolute risk reduction by statin treatment, those who benefit meaningfully from statin therapy in absolute terms in the primary prevention setting can be identified.

Current guidelines recommend statin treatment for the secondary prevention of MACE in general, but where possible treatment decisions should ideally be made per individual taking into account expected absolute treatment effect, adverse events and patient preferences [6–8]. High absolute treatment effects found in our study underline this recommendation, and moreover enable physicians to estimate the individual absolute treatment effect for a patient. There seems to be a maximal absolute risk reduction that can be achieved by a statin in this high-risk population, as a statin may delay recurrent MACE in patients at very high risk (≥70 % 10-year MACE risk) rather than prevent it from happening during a lifetime. In these individuals, the benefit from statin therapy in recurrent MACE-free life years might be limited. In general, treating elderly patients with vascular disease with a statin seems beneficial as the large expected benefits are very likely to outweigh potential harms. These include adverse events like myopathy and incident type 2 diabetes, drug–drug interactions and the inconvenience of polypharmacy which impair quality of life in elderly patients in particular [27, 28]. An example of a drug–drug interaction that increases the risk of adverse events comes from the United States of America where 83 % of patients with dyslipidemia is treated with a CYP3A4-metabolized statin of whom 25–30 % concomitantly use a CYP3A4-inhibitor [29]. Even though there might be a higher risk of serious adverse events in the elderly, there is no conclusive evidence for a higher incidence of rhabdomyolysis, cognitive deterioration, liver or kidney injury [30].

For primary prevention of vascular disease, current guidelines advise to treat those at high risk which means that practically everyone aged ≥70 years would be given a statin since age dominates risk scores [6, 7, 9]. However, in clinical practice statin treatment rates for elderly patients are low presumably reflecting ambiguities about the absolute benefit of statin treatment for the primary prevention of vascular disease in the elderly [11]. Moreover, the incidence of severe comorbidities increases with age and emphasis might be placed on treating these inter-current illnesses. Our prediction model shows that the absolute effect of a statin on MACE is influenced by individual patient characteristics. With the use of this prediction model those individuals who benefit most from statin treatment can be identified. A patient’s advantage of statin therapy in terms of reduction of absolute MACE risk can be estimated and weighed against potential harms of treatment and the costs of statin therapy, even though these are low, in making a treatment decision. A potential harm of statin therapy found in the PROSPER trial was an increase in cancer incidence [4]. However, a meta-analysis of 35 large randomised controlled trials found an equal risk of cancer in those with and without statins [31]. Even so, there was no increased cancer risk in statin users during the extensive 8–11 year follow-up of both the PROSPER and ASCOT-LLA trial [32, 33]. In patients aged >70 years from the ASCOT-LLA population (*n* = 2415), atorvastatin did not raise cancer risk (sHR 0.83, 95 % CI 0.58–1.20). Therefore, it is likely that statin therapy does not increase cancer incidence. For adequate estimations of MACE risk and the absolute risk reduction with a statin, death due to cancer was taken into account as a competing event.

Apart from the estimation of individual absolute statin treatment effects, these models inform physicians and patients about an individual’s 5-year or 10-year risk for MACE. Thereby the need for preventive medical and life-style interventions could be established. Informing patients about their risk and engaging them in treatment decision-making might stimulate treatment adherence [34]. Vascular risk estimation in elderly patients has been challenging and the Systematic Coronary Risk Evaluation (SCORE), QRISK2 and Framingham/Pooled Cohort Equations risk charts are not validated for patients >65 years, >74 years and >79 years of age, respectively [10, 35, 36]. Furthermore, they do not take into account that many elderly patients die from a nonvascular cause. One risk score for patients aged ≥65 years accounted for competing events like the risk score in this study, but that study included only patients without vascular disease and the outcome was coronary artery events instead of MACE as in this study [17]. Interestingly, LDL-cholesterol was a weak predictor for MACE in both patients with and without vascular disease. Other risk factors contribute more to risk prediction in the elderly. As our aim was to predict individual absolute benefit from statin therapy we did not assess causality. Previous studies showed no or an inverse association between LDL-cholesterol and all-cause mortality [37]. In the PROSPER trial pravastatin lowered MACE risk with 15 % whereas it had no effect on all-cause mortality, which implies that the causal association between LDL-cholesterol and MACE may differ from the association between LDL-cholesterol and mortality.

### Strengths and limitations

A strength of this study is that the prediction models were derived and externally validated in an elderly population. Moreover, the variable ‘number of medications’ was added to the models as a proxy for comorbidity [38, 39]. Also, we accounted for competing events (death due to a nonvascular cause) in our statistical analysis. Furthermore, the model for those with vascular disease was validated in a patient population from a cohort study. Thereby, we show that our model is generalizable to a broad elderly population and not restricted to relatively healthy patients in trials. There are some limitations of this study. Overall discriminative ability of these models was moderate, and low in the ASCOT-LLA population. This could be explained by the homogeneity of trial populations in general and of the ASCOT-LLA population in particular with a small range in MACE risk [40]. The adequate calibration of these models may be more important in assessing model validity, as we aim to accurately predict MACE risk and the absolute statin treatment effect for the individual elderly patient. In the PROSPER and ASCOT-LLA trial, fixed statin doses were given and dosing was not titrated to a specific LDL-cholesterol target. Patients in the SMART study used different statins and dosages. It could be that treatment effects for more potent statins or dosages are underestimated with the current model [41]. In sensitivity analyses we established what the individual absolute risk reduction might be with atorvastatin 20 mg or the treatment effect from a meta-analysis in elderly subgroups from statin trials. These results should be interpreted with caution as the meta-analysis was performed in slightly older patients (≥75 years) for an LDL-reduction of 1 mmol/l, for a different vascular outcome and not taking competing risks into account [25]. Finally, our results cannot be extrapolated to the very old (≥85 years) and to patients with chronic kidney disease stage IV or V (eGFR <30 ml/min), since they were not enrolled in these studies.

## Conclusions

A multivariable prediction model can be used to quantify the absolute MACE risk and absolute MACE risk reduction in 5 and 10 years by statin therapy in individual elderly patients with and without vascular disease. Most elderly patients with vascular disease have high predicted absolute MACE risk reduction by a statin and it is most beneficial to treat them all with a statin for secondary prevention of vascular disease. The prediction model identifies the elderly patients who benefit most (i.e., meaningfully in terms of ARR) from statin therapy for primary prevention of MACE. The model could help physicians in managing vascular risk in their elderly patients, a population rapidly rising in prevalence.

## Electronic supplementary material

Below is the link to the electronic supplementary material.

Supplementary material 1 (DOCX 160 kb)
