# Supplementary material for: Personalized absolute benefit of statin treatment for primary or secondary prevention of vascular disease in individual elderly patients
Source: Clin Res Cardiol. 2016 Aug 23;106(1):58–68. doi: 10.1007/s00392-016-1023-8 (PMC5226996; doi:10.1007/s00392-016-1023-8)
Supplement: Supplementary file 1 — Supplementary material 1 (DOCX 160 kb) [file 392_2016_1023_MOESM1_ESM.docx]

**Supplementary material**

**Personalized absolute benefit of statin treatment for primary or secondary prevention of vascular disease in individual elderly patients

Clinical Research in Cardiology**

Manon C. Stam-Slob; Frank L.J. Visseren*; J. Wouter Jukema; Yolanda van der Graaf; Neil R. Poulter; Ajay Gupta, Naveed Sattar; Peter W. Macfarlane, Patricia M. Kearney; Anton J.M. de Craen; Stella Trompet
* Corresponding author. University Medical Center Utrecht, Utrecht, the Netherlands.
E-mail address: [f.l.j.visseren@umcutrecht.nl](mailto:f.l.j.visseren@umcutrecht.nl)

**Content**

Table S1 (Model formula) – page 2
Methods and calculation example Net Benefit – page 3
Fig. S1 (Net benefit) – page 4
Table S2 (Net benefit) – page 4

**Table S1. Estimation of MACE risk and treatment effect of statin for individual elderly patients**

**Risk estimation for patients with vascular disease**

| Individual risk for MACE (%) | 5-year: | 10-year: |
| --- | --- | --- |
| Scotland / Ireland: | (1 - 0.727 ^^exp(^**^A^** ^– 2.389)^) * 100% | (1 - 0.529 ^^exp(^**^A^** ^– 2.389)^) * 100% |
| Rest of North- and West-Europe: | (1 - 0.828 ^^exp(^**^A^** ^– 2.359)^) * 100% | (1 - 0.644 ^^exp(^**^A^** ^– 2.359)^) * 100% |

**A** = linear predictor for both 5- and 10-year risk = 0.401 (if male) + 0.042 * (age in years) + 0.240 (if current smoker) + 0.543 (if diabetes) + 0.344 (if polyvascular disease) + 0.053 * (number of medications) - 0.037 * (systolic blood pressure in mmHg) + 0.00012 * (systolic blood pressure in mmHg)^2^ + 0.876 * (LDL-cholesterol in mmol/L) - 0.109 * (LDL-cholesterol in mmol/L)^2^ + 0.081 * (HDL-cholesterol in mmol/L) - 0.0053 * (MDRD in ml/min/1.73) - *0.245 (if treated with a statin)*

**Risk estimation for patients without vascular disease**

| Individual risk for MACE (%) | 5-year: | 10-year: |
| --- | --- | --- |
| Scotland / Ireland: | (1 - 0.838 ^^exp(A – 1.934)^) * 100% | (1 - 0.703 ^^exp(A – 1.934)^) * 100% |
| Rest of North- and West-Europe: | (1 - 0.895 ^^exp(A – 1.968)^) * 100% | (1 - 0.801 ^^exp(A – 1.968^) * 100% |

**A** = linear predictor for both 5- and 10-year risk = 0.283 (if male) + 0.037 * (age in years) + 0.290 (if current smoker) + 0.210 (if diabetes) + 0.090 * (number of medications) + 0.0060 * (systolic blood pressure in mmHg) + 0.0070 * (LDL-cholesterol in mmol/L) - 0.359 * (HDL-cholesterol in mmol/L) - 0.061 * (MDRD in ml/min/1.73) + 0.00048 * (MDRD in ml/min/1.73) ^2^ - *0.140 (if treated with a statin)*

**Absolute risk reduction (ARR)**

ARR = individual MACE risk (%) without a statin – individual MACE risk (%) with a statin

**Calculation example Net Benefit for a 5-year NWT = 40**

The net benefit method by Vickers et al.[^1^](#_ENREF_1) is an instrument for weighing benefit and harms of treatment for different treatment strategies on a group level. Benefit in the measure is the observed mean absolute risk reduction in the population. Harm is not measured directly, but includes expected costs, side effects and other negative effects of treatment. Net benefit is presented in a graph for different treatment thresholds. A treatment threshold is chosen by a physician who decides how many patients he or she is willing to treat to prevent one event, i.e. the number willing to treat (NWT). The treatment threshold is the minimal absolute risk reduction for which a physician believes the benefit of statin treatment outweighs the harm of treatment (= 100/NWT). For example, a 5-year NWT of 50 corresponds to a treatment threshold of 2%, which means that only patients with a predicted 5-year ARR ≥2% are treated with a statin. Harm is indirectly incorporated in the treatment threshold, as one will only treat patients who have high absolute treatment effect if a treatment is considered harmful (low NWT). If a treatment is relatively safe and cheap, both patients with high and lower absolute treatment effect may be treated, which corresponds to a low treatment threshold (high NWT).

Now we provide an example of net benefit estimation for a statin in elderly patients without vascular disease. In figure 3B, net benefit is shown for a range of treatment thresholds. In this example, a NWT for 5 years of 40 is chosen, which corresponds to a treatment threshold ARR of 2.5% (=100/40).

|  | **Treat none** | **Treat all** | **Prediction-based treatment** |
| --- | --- | --- | --- |
| N patients treated (%) | 0 (0%) | 5146 (100%) | 693 (13.47%) |
| 5-year event rate | 15.91% | 13.45% | 14.98% |
| Decrease in 5-year event rate | 0% | 15.91 - 13.45 = 2.46% | 15.91 – 14.98 = 0.93% |
| Net benefit | 0% | -0.04% | 0.59% |

The event rate for the strategy “treat none” is the observed event rate in patients not treated with a statin (15.91%). For the strategy “treat all”, the event rate is the observed event rate in patients treated with a statin (13.45%). If all patients are treated with a statin, 2.46% less major cardiovascular events will occur compared to treating none.

The event rate for prediction-based treatment is the observed event rate in patients treated with a statin for whom the prediction model recommends treatment (predicted ARR ≥2.5%), plus the observed event rate in patients not on a statin for whom the prediction model discourages treatment (predicted ARR <2.5%). The event rate for prediction-based treatment is 14.98%, which means a 0.93% decrease in 5-year event rate compared to treating none.

Net benefit is estimated as follows:

| Decrease in 5-year event rate (%) - | (Patients treated (%) * | Treatment threshold (=1/NWT)) | |
| --- | --- | --- | --- |
| *Benefit* | *Harms* | |  |

Net benefit treat all = 2.46% - (100% * (1/40)) = -0.04%

Net benefit prediction-based treatment = 0.93% - (13.47% * (1/40)) = 0.59%

This means that if one chooses a NWT of 40, treatment according to the prediction model has a more favorable trade-off between benefit and harms of treatment than treating all patients.

**Fig. S1 Net benefit analysis**

**
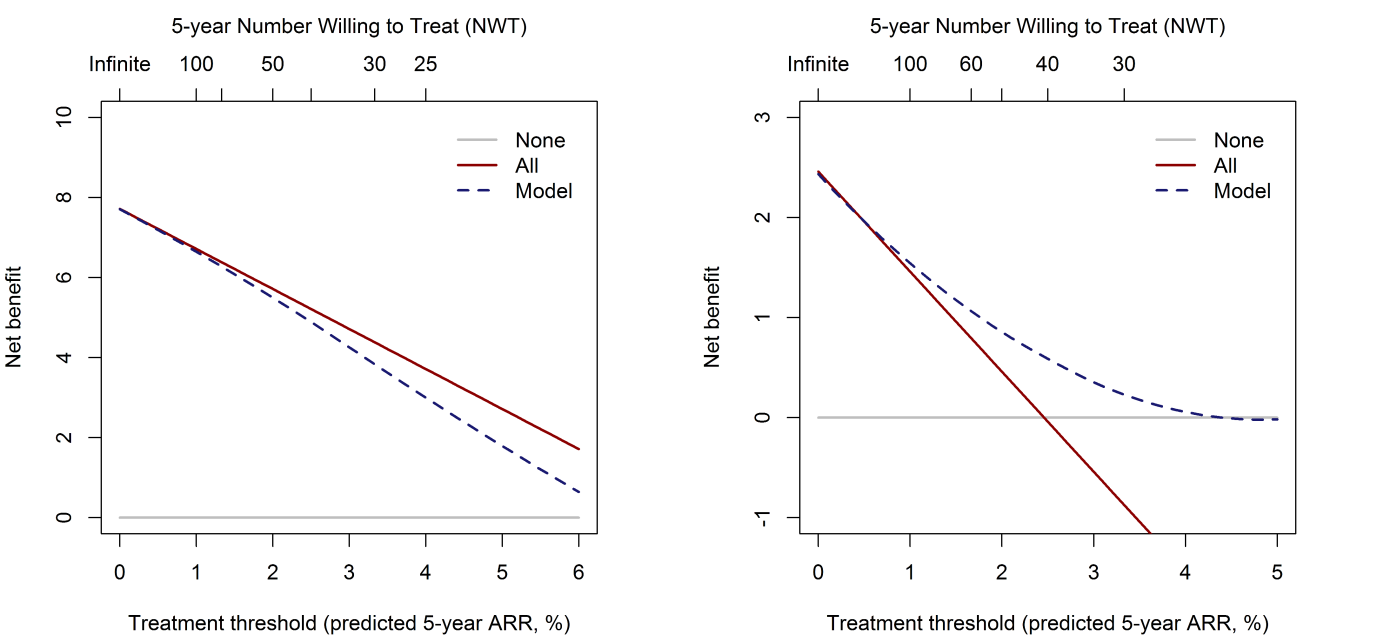
A - Patients with vascular disease B - Patients without vascular disease**

For secondary prevention of vascular disease, treating all elderly patients with a statin is more beneficial than prediction-based treatment irrespective of the treatment threshold a physician chooses. For primary prevention of vascular disease, treatment according to the prediction model is more beneficial than treating all elderly patients with a statin for a treatment threshold ≥1.0% predicted 5-year ARR (5-year NWT ≤100).

| **Table S2. Net benefit analysis in patients without vascular disease (eFigure 1B)** | | | | |
| --- | --- | --- | --- | --- |
| *NWT* | *Strategy with  highest net benefit* | *Patients treated (%)* | *Event rate (%)** |  |
| Infinite (>100) | Treat all | 100 | 13.5 |  |
| 100 | Prediction model | 93 | 13.5 |  |
| 60 | Prediction model | 51 | 14.5 |  |
| 50 | Prediction model | 31 | 14.6 |  |
| 40 | Prediction model | 13 | 15.0 |  |
| ≤ 30 | None | 0 | 15.9 |  |
| **Event rate as the percentage of patients that experience MACE, regardless of their treatment allocation* | | | | |

**Reference**

**1.** Vickers AJ, Kattan MW, Daniel S. Method for evaluating prediction models that apply the results of randomized trials to individual patients. *Trials.* 2007;8:14.
